# Who does what the cardiologist recommends? Psychosocial markers of unhealthy behavior in coronary disease patients

**DOI:** 10.1371/journal.pone.0228262

**Published:** 2020-01-31

**Authors:** Mercedes Arrebola-Moreno, Dafina Petrova, María-José Sánchez, Ricardo Rivera-López, José Antonio Ramírez-Hernández

**Affiliations:** 1 Mind, Brain and Behavior Research Center (CIMCYC), University of Granada, Granada, Spain; 2 Escuela Andaluza de Salud Pública, Granada, Spain; 3 Instituto de Investigación Biosanitaria ibs.GRANADA, Granada, Spain; 4 CIBER of Epidemiology and Public Health (CIBERESP), Madrid, Spain; 5 University of Granada, Granada, Spain; 6 Cardiology Department, Virgen de las Nieves University Hospital, Granada, Spain; University of North Texas Health Science Center, UNITED STATES

## Abstract

Patients diagnosed with coronary heart disease should follow lifestyle recommendations that can reduce their cardiovascular risk (e.g., avoid smoking). However, some patients fail to follow these recommendations and engage in unhealthy behavior. With the aim to identify psychosocial factors that characterize patients at high risk of repeated cardiovascular events, we investigated the relationship between social support, mental health (coping, self-esteem, and perceived stress), and unhealthy behavior. We conducted a cross-sectional study of 419 patients recently diagnosed with coronary heart disease (myocardial infarction or angina) who participated in the National Health Survey in Spain (2018). Unhealthy behaviors were defined according to the European Guidelines on cardiovascular disease prevention. Only 1% of patients reported no unhealthy behaviors, with 11% reporting one, 40% two, 35% three, and 13% four or more unhealthy behaviors. In multiple regression controlling for demographic and traditional risk factors, mental health was the only significant psychosocial factor, doubling the odds of accumulated unhealthy behaviors, *OR*(high vs. low) = 2.03, 95% CI [1.14, 3.64]. Mental health was especially strongly related to unhealthy behavior among patients with obesity, *OR*(high vs. low) = 3.50, 95% CI [1.49, 8.45]. The relationship between mental health and unhealthy behaviors suggests that a large proportion of patients may not adhere to lifestyle recommendations not because they purposefully choose to do so, but because they lack coping skills to maintain the recommended healthy behaviors. Low mental well-being may be especially detrimental for behavior change of patients with obesity.

## Introduction

Cardiovascular diseases (CVD) are the leading cause of death in Europe [1]. Globally, in 2016 they accounted for 31% of all deaths worldwide [2]. CVDs are also the leading cause of death in Spain, with coronary heart diseases representing 105 deaths (154 in men and 69 in women) per 100,000 inhabitants, according to the latest data of the National Statistics Institute (2017) [3].

The burden of coronary heart disease (CHD) can be dramatically reduced through prevention efforts targeting healthier lifestyle. In particular, healthy lifestyle could help prevent and control hypertension, high cholesterol, obesity, or diabetes, all of which significantly increase the risk of CHD [4]. The prevention of future cardiac events and complications in patients already diagnosed with CHD is an important policy focus because these patients are at 5–6 times higher risk of suffering cardiovascular events [4]. To reduce this risk, patients are advised to follow certain health recommendations such as to engage in moderate-intensity physical activity, to eat a healthy diet, maintain a healthy weight, not to smoke, and not to drink large amounts of alcohol [4]. However, research in 24 European countries shows that, although many patients try to follow the recommendations regarding lifestyle, compliance is not satisfactory and objectives are frequently not achieved [5]. Similarly, less than half of eligible patients in Europe benefit from cardiac rehabilitation programs [6]. Engaging in behaviors that increase the risk of repeated cardiac events (e.g., smoking or drinking alcohol) or not engaging in highly recommended behaviors that could reduce this risk (e.g., exercising, eating a healthy diet) reflects what we refer to as a pattern of unhealthy behaviors. A patient who does not follow a larger number of recommendations exposes him- or herself to larger cardiovascular risk.

Past research has identified several demographic predictors of unhealthy behavior. For instance, several studies have suggested that men follow medical recommendations to a lesser extent than women [7,8], whereas married patients follow recommendations to a larger extent than unmarried patients [9].

Besides demographic factors, psychosocial factors can also influence the risk of subsequent cardiac events and are likely more proximal predictors of behavior that drive the effects of socio-demographic factors [10,11]. Some of the psychosocial factors that have been related to increased cardiovascular risk are: lack of social support [12], low resilience [13], stress [10], depression [14], anxiety [15], and type-D personality [16]. Part of the effect of psychosocial factors on cardiovascular risk could be driven by their influence on behavior. To illustrate, patients with low social support are less likely to engage in cardiovascular risk screening [17] and have more trouble with the maintenance of self-care behaviors to manage their disease [18].

In the current research, we investigated the relationship of two psychosocial factors–social support and mental health–with unhealthy behaviors in CHD patients. Low social support has been associated with a higher risk of cardiac mortality [12,19] and a greater exposure to behavioral risk factors in healthy populations [20, 21]. Mental health problems such as depression are especially likely to develop following a cardiac event and have been associated with a two-fold increase in the risk of negative cardiovascular outcomes (e.g., mortality) [22]. Depressive symptoms have also been linked to lower adherence to risk-reducing health behaviors [23,24] and lower medication adherence [25]. Both lack of social support from friends and family and depression have been associated with a lower likelihood of taking up and completing cardiovascular lifestyle behavior change programs [26].

Guidelines for rehabilitation and self-care of CHD patients place emphasis on addressing the full range of modifiable risk factors [27]. However, no study to our knowledge has examined the relationships of social support and mental health with the global unhealthy behavior profile of patients. Because social support has also been associated with depression in cardiac patients [28], it is possible that its beneficial effects on health-related behavior are explained by mental health; thus, it will be important to investigate the independent relationships of these two factors with unhealthy behavior. In addition, most previous studies investigated these relationships in healthy populations [20,21] or focused specifically on the presence of clinical depression [23]. Using a broader measure of mental health can capture manifestations of different types of mental distress and provide indication as to what specific cognitive processes (e.g., problems with coping or self-esteem) could be associated with unhealthy behavior.

To fill this gap, we investigated the relationships of social support and general mental health with unhealthy behaviors in a sample of CHD patients drawn from a representative sample of the Spanish population. In particular, we aimed to describe the type and number of unhealthy behaviors reported by these patients, and quantify the unique relationships of social support and mental health with the number of unhealthy behaviors. The identification of psychosocial markers of unhealthy behavior could help identify patients who need intervention to prevent new cardiovascular events. It could also reveal potential underlying mechanisms that hinder the successful reduction of cardiovascular risk among this vulnerable group and thus provide recommendations regarding intervention design.

## Method

We conducted a cross-sectional descriptive study using survey data from the latest National Health Survey (NHS), conducted in 2017–2018 by the Spanish Ministry of Health, Social Services and Equality, and the National Institute of Statistics of Spain. The survey covered the entire territory of Spain and multistage stratified random sampling was used to obtain a representative sample of the adult population [29]. Data were collected through a personal computer-assisted interview by trained interviewers. The number of households selected was 37,500 and these were distributed in 2,500 census sections; the census sections were grouped into six strata, according to the size of the municipality to which they belonged and were selected with a probability proportional to this size. Fifteen households were randomly selected within each census section. From each household, one adult was selected at random to participate in the survey. The response rate was 95% and responses were gathered from 23,090 adults.

For the current research, we selected those respondents who: a) were 40 years old or older at the time of the survey because the prevalence of CHD increases after this age [30] and b) reported that they were diagnosed with CHD (acute myocardial infarction or angina) in the last 12 months. A total of N = 419 people met these criteria: 150 (36%) reporting myocardial infarction (MI) only, 235 (56%) reporting angina only, and 34 (8%) reporting both. No ethical approval was required for this research as it involved secondary data analysis. The data and code for the analyses are available on the Open Science Framework (doi: 10.17605/OSF.IO/3D9FA).

### Measures

#### Number of unhealthy behaviors

Participants answered various questions about their health-related behavior from which we extracted data regarding behaviors considered as modifiable behavioral risk factors for coronary heart disease based on the guidelines of the European Society of Cardiology (ESC) [4]. For each participant, we recorded whether he/she reported behavior that did not comply with the guidelines of the ESC regarding physical activity, alcohol consumption, smoking, vegetable, fruit, fish, sugary drinks, and fast food consumption. We assigned participants a score of one for each behavior not in line with recommendations and summed up the total number of behaviors (0 to 8); occasional survey non-responses (e.g., “do not know”, <1%) were coded as not in line with recommendations. Regarding physical activity, patients were regarded as completing recommendations when they reported either (a) doing physical activity in their leisure time at least several times a week or (b) reported frequent physical activity during their principal daily activity (e.g., work) such as walking, carrying weight, moving frequently, or doing tasks that require great physical effort. The ESC recommends that individuals accumulate at least 150 min/week of moderate intensity physical activity or 75 min/week of vigorous intensity physical activity; however, in the survey physical activity was assessed in such detail only in individuals younger than 70. Thus, we created a proxy criterion based on the available information. Regarding alcohol consumption, unhealthy behavior was defined as more than 20 gr/day of pure alcohol on average for men and 10 gr/day for women as per ESC recommendation. Alcohol consumption data were based on a variable derived from an extensive assessment incorporated in the survey in which participants were asked about their consumption of various alcoholic drinks and the mean consumption of pure alcohol in grams per week was derived. Regarding smoking, unhealthy behavior was defined as currently smoking (daily or not). Regarding diet, the following consumption was regarded as unhealthy behavior as per ESC guidelines: fewer than two servings of vegetables daily, fewer than two servings of fruit daily, fish consumption less than once/twice a week. ESC guidelines discourage the consumption of sugar-sweetened soft processed food rich in saturated fat, thus three or more times a week consumption of fast food (e.g., fried chicken, sandwiches, pizzas, hamburgers) and sugary drinks was regarded as unhealthy behavior. The questions about diet were based on multiple choice items (all available on the statistical portal of http://www.msssi.gob.es).

#### General Health Questionnaire (GHQ-12)

To measure mental health, the validated Spanish version of this instrument was administered [31]. The instrument is designed to screen for general non-psychotic psychiatric morbidity using questions measuring problems with coping, low self-esteem, and perceived stress [31]. In particular, twelve questions assess participants’ mental health in the past two weeks compared to usually on scales from 0 to 3, where a higher score indicates worse mental health. We used the sum of the scores on all items (Cronbach’s alpha = .93) as a measure of mental health.

#### Social support

This was measured with the Spanish version of the Functional Social Support Questionnaire of Duke-UNC [32]. This instrument includes 11 items measuring perceived emotional and instrumental support on scales from 1 "much less support than I want" to 5 "as much support as I want". The final score (Cronbach’s alpha = 0.93) ranges between 11 and 55, where higher scores indicate higher perceived social support.

#### Socio-demographic variables

We recorded participants’ age, gender, marital status (married, single, widowed, separated/divorced), and social class (based on the classification of Domingo-Salvany [33]; category descriptions are found in Table 1).

**Table 1:** Descriptive statistics (number of cases and percentage from total) for categorical variables used in the study.

		All patients		Myocardial infarction		Angina	
Variable	Categories	N	419	N	184	N	269
Gender	Man	233	56%	112	61%	144	54%
	Woman	186	44%	72	39%	125	46%
Social Classal	Category I: Directors and managers of establishments of 10 or more employees and professionals traditionally associated with university degrees.	27	6%	12	7%	18	7%
	Category II: Directors and managers of establishments of less than 10 workers, professionals traditionally associated with university diplomas and other technical support professionals. Sportsmen and artists.	15	4%	6	3%	10	4%
	Category III: Intermediate occupations and self-employed workers.	70	17%	29	16%	46	17%
	Category IV: Supervisors and workers in qualified technical occupations.	62	15%	32	17%	35	13%
	Category V: Qualified workers from the primary sector and other semi-skilled workers.	160	38%	66	36%	109	41%
	Category VI: Unskilled workers.	65	16%	31	17%	39	14%
	Missing	20	5%	8	4%	12	4%
Civil Status	Single	41	10%	15	8%	29	11%
	Widowed	114	27%	45	24%	78	29%
	Separated/divorced	26	6%	13	7%	16	6%
	Married	238	57%	111	60%	146	54%
Diabetes	Yes	137	33%	55	30%	98	36%
	No	282	67%	129	70%	171	64%
Hypertension	Yes	284	68%	127	69%	183	68%
	No	135	32%	57	31%	86	32%
High cholesterol	Yes	246	59%	108	59%	158	59%
	No	173	41%	76	41%	111	41%
Depression	Yes	97	23%	37	20%	69	26%
	No	321	77%	146	79%	200	74%
	Missing	1	0%	1	1%	0	0%
Chronic anxiety	Yes	66	16%	26	14%	48	18%
	No	352	84%	157	85%	221	82%
	Missing	1	0%	1	1%	0	0%
Body-mass index (BMI)	Underweight (< 18,5 kg/m^2^)	6	1%	4	2%	2	1%
	Normal weight (18,5 kg/m^2^ ≤IMC < 25 kg/m^2^)	106	25%	50	27%	63	23%
	Overweight (25 kg/m^2^ ≤ IMC < 30 kg/m^2^)	175	42%	72	39%	119	44%
	Obesity (≥30 kg/m^2^)	111	26%	44	24%	76	28%
	Missing	21	5%	14	8%	9	3%
Unhealthy behaviors	None	4	1%	1	1%	3	1%
	1	45	11%	21	11%	30	11%
	2	167	40%	61	33%	113	42%
	3	148	35%	70	38%	93	35%
	4	42	10%	22	12%	25	9%
	5	7	2%	7	4%	1	0%
	6	5	1%	1	1%	4	1%
	7	1	0%	1	1%	0	0%
Categories of unhealthy behaviors	Low: None or one	49	12%	22	12%	33	12%
	Low-medium: Two	167	40%	61	33%	113	42%
	Medium-high: three	148	35%	70	38%	93	35%
	High: four or more	55	13%	31	17%	30	11%
Physical activity	Active/within norm	51	12%	24	13%	32	12%
	Inactive	368	88%	160	87%	237	88%
Alcohol consumption	Within norm (≤20 gr/d for men and ≤10 gr/d for women)	400	95%	175	95%	259	96%
	High	19	5%	9	5%	10	4%
Smoking	Does not smoke	377	90%	165	90%	244	91%
	Smokes	42	10%	19	10%	25	9%
Vegetable consumption	Within norm	44	11%	20	11%	29	11%
	Low (< two servings daily)	375	89%	164	89%	240	89%
Fruit consumption	Within norm	239	57%	97	53%	157	58%
	Low (< two servings daily)	180	43%	87	47%	112	42%
Fish consumption	Within norm	383	91%	160	87%	250	93%
	Low (less than once/twice a week)	36	9%	24	13%	19	7%
Sugary drinks	Within norm	386	92%	163	89%	253	94%
	High (three or more times a week)	33	8%	21	11%	16	6%
Fast foods	Within norm	409	98%	179	97%	264	98%
	High (three or more times a week)	10	2%	5	3%	5	2%

#### Further CHD risk factors

We recorded whether patients had at any point suffered diabetes, hypertension, high cholesterol, chronic anxiety, and depression. Patients’ body-mass index (BMI) was calculated based on self-reported weight and height and obesity was defined as BMI>30 kg/m^2^. Finally, we computed the total number of comorbidities reported, i.e., number of other diseases that the patient reported having suffered (e.g., cancer, asthma, rheumatism, osteoporosis, etc.) from a total of 24 possible diseases, as other diseases and their cumulative impact could also be related to participants’ health-related behavior.

### Analysis

The dependent variable of interest was the number of unhealthy behaviors. The predictors of main interest were the psychosocial factors: mental health and social support. The rest of the variables were regarded as control variables. First, we used descriptive statistics to summarize the general characteristics of the sample and the most common unhealthy behaviors. Then we analyzed the relationship between the psychosocial factors and the unhealthy behaviors. We first conducted simple regressions with each psychosocial factor as predictor and unhealthy behaviors as dependent variable. We finally conducted a multiple regression analysis including the two psychosocial factors and the demographics and traditional risk factors. The rate of missing data was low and no imputation was performed.

In the case of categorical predictors of more than two categories (social class and marital status), the following categories were used as references in comparisons between the different levels: the lowest social class (VI) and being married. These categories have been previously associated with cardiovascular risk (a higher risk in the case of low social class and a protective effect in the case of being married (9,19)). The odds ratio (OR) was used as an estimator of the effect size and 95% confidence intervals were computed to determine the significance of the predictors (intervals excluding 1). The analyses were carried out using the packages *summarytools* and *MASS* (function polr) in *R*.

## Results

Table 1 shows descriptive statistics for the categorical variables and Table 2 shows descriptive statistics for the continuous measures for all patients and based on type of CHD (MI or angina). Only 1% of patients reported no unhealthy behaviors, with 11% reporting one, 40% two, 35% three, and 13% four or more unhealthy behaviors. Patients with MI reported more unhealthy behaviors compared to patients with angina, OR = 1.48, 95% CI [1.04, 2.13]. Regarding the specific behaviors (see Table 1), the proportion of patients reporting behavior not consistent with recommendations was highest for vegetable consumption (89%) and physical activity (88%), followed by fruit consumption (43%), smoking (10%), fish (9%), and sugary drinks consumption (8%). Only a small proportion of patients did not adhere to recommendations regarding alcohol consumption (5%) and fast foods consumption (2%).

**Table 2:** Descriptive statistics (minimum, maximum, mean and standard deviation) for the continuous variables used in the study (N = 419).

		Minimum	Maximum	Median	Mean	Standard deviation	% Missing data
All patients (N = 419)	Age (years)	40	97	73	72.0	11.7	0%
	Comorbidities (n°)	0	6	2	2.6	1.7	0%
	Mental health	1	36	11	13.2	6.8	1%
	Social Support (Duke-UNC)	11	55	49	47.1	8.4	6%
Myocardial infarction (N = 184)	Age (years)	43	95	73	70.7	11.8	0%
	Comorbidities (n°)	0	6	2	2.5	1.7	0%
	Mental health	1	36	12	13.7	7.1	0%
	Social Support (Duke-UNC)	18	55	49	47.0	8.0	7%
Angina (N = 269)	Age (years)	40	97	74	72.6	11.5	0%
	Comorbidities (n°)	0	6	2	2.6	1.7	0
	Mental health	4	36	11	13.1	6.6	1%
	Social Support (Duke-UNC)	11	55	49	47.0	8.8	8%

Because not many individuals reported either zero or four or more unhealthy behaviors, an ordinal variable “Categories of unhealthy behavior” was created (see Table 1) and regression analyses were conducted on this ordinal variable. In particular, we first conducted simple ordinal logistic regressions (*polr* in *R*). Higher social support was related to fewer unhealthy behaviors (albeit insignificantly), *OR* = .98, 95% CI [.96, 1.00]. Worse mental health was related to more unhealthy behaviors, *OR* = 1.03, 95% CI [1.002, 1.06].

**Fig 1:**
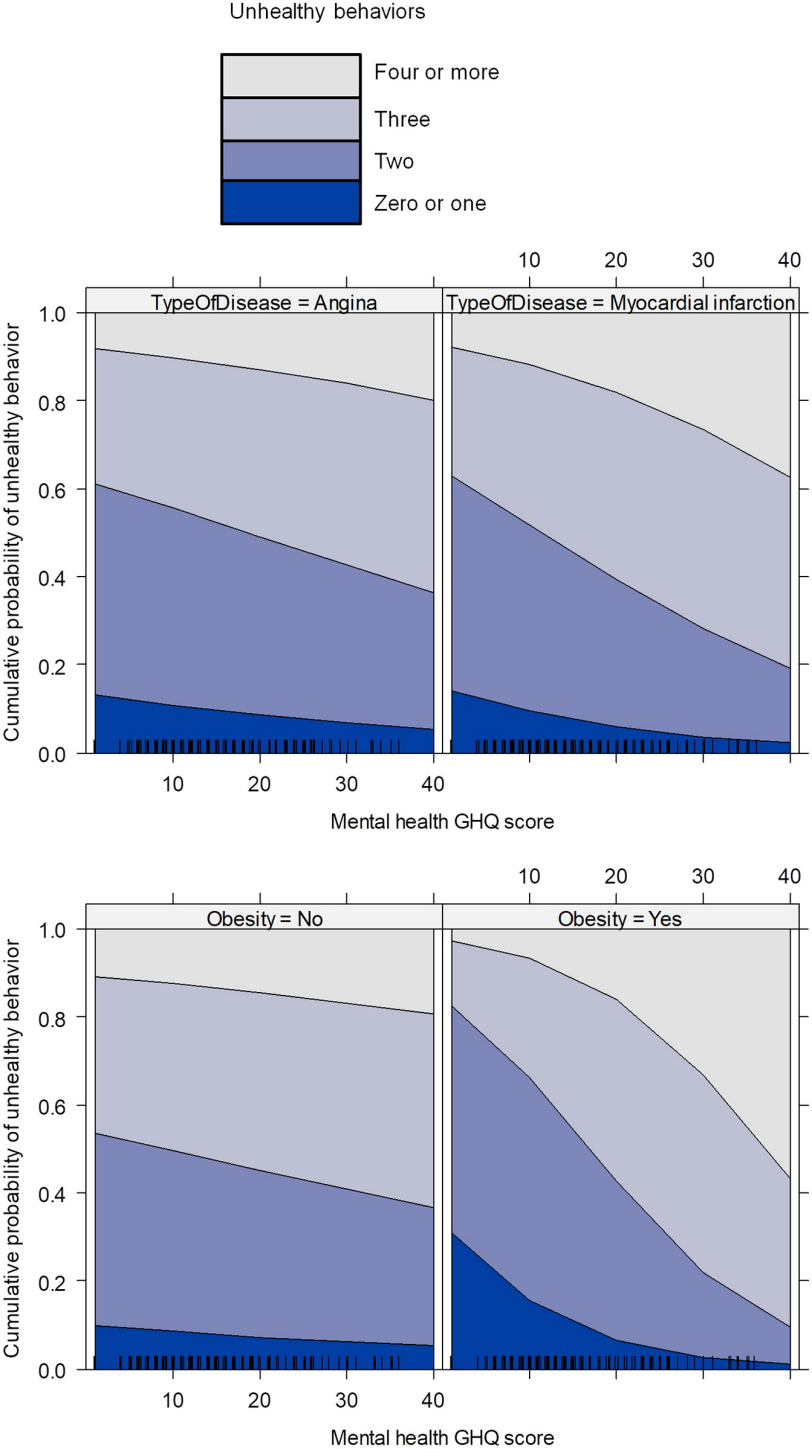
Relationship between mental health and the cumulative predicted probability of unhealthy behavior for each category derived from the multiple ordinal regression model, as a function of type of disease and the presence of obesity.

We then conducted multiple regression analysis, the results of which are shown in Table 3 and Fig 1. Mental health remained significant in the multiple regression model, *OR* = 1.04, 95% CI [1.002, 1.08]. To further illustrate its effect, we divided the mental health variable into terciles and conducted the same multiple regression analysis. Both the middle, *OR* = 1.72, 95% CI [1.06, 2.79] and the highest tercile, *OR* = 2.03, 95% CI [1.14, 3.64] were related to more unhealthy behaviors compared to the lowest tercile.

**Table 3:** Results from multiple ordinal logistic regression.

Predictor	*B*	*SE*			95% Confidence Interval	
			*t*-value	Odds ratio	Inferior	Superior
Gender [female vs. male]	-0.65	0.24	-2.72	**0.524**	**0.328**	**0.834**
Social class (medium vs. low)	-0.32	0.22	-1.41	0.728	0.468	1.129
Social class (high vs. low)	-0.14	0.34	-0.42	0.867	0.445	1.681
Civil status (separated/divorced/single vs. married)	-0.06	0.30	-0.20	0.944	0.528	1.686
Civil status (widowed vs. married)	0.43	0.28	1.54	1.544	0.890	2.686
Age	-0.03	0.01	-2.67	**0.973**	**0.954**	**0.993**
Diabetes (yes vs. no)	0.16	0.23	0.70	1.175	0.749	1.843
Hypertension (yes vs. no)	0.30	0.23	1.30	1.354	0.857	2.142
High cholesterol (yes vs. no)	-0.12	0.22	-0.57	0.883	0.575	1.355
Obesity (yes vs. no)	-0.45	0.23	-1.96	**0.641**	**0.410**	**0.997**
Chronic anxiety (yes vs. no)	-0.31	0.34	-0.91	0.734	0.376	1.424
Depression (yes vs. no)	-0.19	0.30	-0.65	0.825	0.458	1.479
Comorbidities	0.01	0.06	0.10	1.006	0.889	1.138
Mental health	0.04	0.02	2.05	**1.038**	**1.002**	**1.075**
Social support	-0.01	0.01	-1.04	0.986	0.960	1.013
Type of disease (MI vs. angina)	0.22	0.21	1.05	1.242	0.827	1.867

Significant effects (95% confidence intervals for the odds ratio excluding 1) are marked in bold. B = unstandardized regression coefficient. SE = standard error of B. For social class, the original six categories were grouped into high (I and II), medium (III and IV), and low (V and VI) due to low cell counts in some of the categories. For civil status, the category “single” was grouped with “separated/divorced” due to low cell count.

Among the control variables, women, OR = 0.52, 95% CI [0.33, 0.83], younger patients, OR = 0.97, 95% CI [0.95, 0.99], and patients with obesity, OR = 0.64, 95% CI [0.41, 0.99] reported fewer unhealthy behaviors. We next checked for significant interactions between mental health and these variables. There were no significant interactions between mental health with gender or age and stratified analysis showed that there were no notable differences in the relationship between mental health and unhealthy behavior across these categories and as a function of type of disease (see Table 4). However, there was a significant interaction, OR = 1.08, 95% CI [1.01, 1.16], between mental health and obesity, showing that mental health was more strongly related to unhealthy behavior among patients who suffer obesity vs. those who do not (see Fig 1 and Table 4). In particular, patients with obesity who reported good mental health (low GHQ-12 scores) were at lower risk of unhealthy behaviors compared to patients without obesity; however, for patients who reported mental health problems (high GHQ-12 scores) this tendency reversed and patients with obesity were at higher risk of unhealthy behavior compared to patients without obesity (Fig 1).

**Table 4:** Relationship between mental health scores and unhealthy behaviors from simple regression analyses as a function of type of CHD, gender, age group, and the presence of obesity.

			Mental health (terciles)					
		N	Medium vs. Low			High vs. Low		
			OR	LLCI	ULCI	OR	LLCI	ULCI
Type of coronary heart disease	MI	184	1.946	0.999	3.821	**1.896**	**1.020**	**3.552**
	Angina	269	1.198	0.709	2.026	**2.020**	**1.156**	**3.551**
Gender	Men	233	1.534	0.882	2.677	**1.998**	**1.100**	**3.656**
	Women	186	1.766	0.895	3.514	**2.022**	**1.034**	**3.995**
Age	40–59	75	2.613	0.904	7.779	2.359	0.893	6.415
	60–80	220	1.486	0.825	2.687	1.522	0.830	2.798
	81+	124	1.419	0.643	3.153	2.158	0.925	5.120
Obesity	No obesity (BMI <30 kg/m^2^)	287	1.452	0.881	2.400	1.347	0.791	2.298
	Obesity (BMI ≥30 kg/m^2^)	111	1.770	0.726	4.358	**3.496**	**1.490**	**8.446**

Comparisons are based on terciles of mental health.

## Discussion

Consistent with previous studies, we observed that patients reporting more unhealthy behaviors tended to have lower levels of social support [19]. We also observed that patients who engaged in more unhealthy behaviors reported worse mental health. However, in multiple regression controlling for demographics and traditional risk factors, mental health emerged as the only significant psychosocial predictor of unhealthy behavior. This suggests that the beneficial effect of social support on reducing unhealthy behavior after a cardiac diagnosis observed in previous studies [26] may be at least partly attributable to the protective effects of social support on mental health [28,34].

We further found that mental health was more strongly related to unhealthy behaviors in patients suffering from obesity. In particular, at scores signifying good mental health, patients with obesity reported fewer unhealthy behaviors than patients without obesity. This finding may reflect these patients’ higher motivation to reduce their high cardiovascular risk. However, at scores signifying the presence of mental health problems, patients with obesity reported more unhealthy behaviors compared to patients without obesity. This reversal shows that mental well-being may be especially important for behavior change in this highly vulnerable population. Unfortunately, European surveys show that about one in two patients with obesity report not having taken any action to lose weight after their coronary event [5].

The documented relationship between mental health and unhealthy behaviors has two important practical implications. On one hand, it suggests that a formal assessment with the GHQ-12 [31] or an informal assessment by a physician or a nurse using open-ended questions [11], could successfully identify CHD patients who might have greater difficulty in carrying out the recommendations regarding lifestyle. On the other hand, these results also suggest that a substantial proportion of patients remain at high cardiovascular risk not because they decide to ignore the physicians’ recommendations, but because they may lack the emotional capacity and coping skills to see these through. The GHQ-12 questionnaire is composed of items measuring problems with coping, low self-esteem, and perceived stress [31], suggesting that these issues could be contributing to unhealthy behavior patterns, especially among patients with obesity. This suggestion is in line with research on cardiac rehabilitation attendance showing that besides beliefs (e.g., the belief that the disease cannot be controlled or has no severe consequences) [35,36], additional barriers such as physical (e.g., lack of transportation) and personal (e.g., embarrassment) barriers [37] deter patients from participation in rehabilitation. Importantly, such external barriers could be potentially overcome with the appropriate coping skills. For instance, in patients with obesity lowering cardiovascular risk, and specifically weight loss, remains a challenge. The current results are in line with the importance of implementing behavioral weight loss (BWL) in cardiac rehabilitation programs [38], as this approach addresses several psychological obstacles to weight loss by using positive reinforcement, self-monitoring, and goal setting, among others.

The current results also contribute to the findings of a recent study showing that Chinese CHD patients with lower self-esteem engaged in fewer health promoting behaviors; in particular, the relationship between self-esteem and behavior was partially mediated by confrontation coping, or the tendency to take direct action regarding stressors [39]. In addition, patients with lower self-esteem showed higher levels of avoidance and resignation coping (i.e., avoiding stressors or not doing anything about them). In the context of the current study, these results support the idea that low self-esteem and the inability to cope with problems and stressors could be preventing coronary heart disease patients from following physicians’ recommendations.

Previous research shows that interventions based on Self-Regulation Theory, involving components such as goal setting, self-monitoring, planning, and feedback can be successful at increasing adherence to lifestyle changes in programs for patients with CHD [40]. For instance, in one study participants who formed action plans about when, where, and how they would exercise, and coping plans about how they would overcome anticipated barriers engaged in more physical exercise two months after discharge from cardiac rehabilitation [41]. Making action and coping plans was also shown to reduce depressive symptoms a year after the intervention, and this effect was mediated by perceived goal attainment [42]. However, these positive effects were observed in patients attending cardiac rehabilitation programs. It remains for future research to establish to what extent such strategies can be helpful in more diverse samples of patients or as part of brief, practical interventions integrated into clinical practice (e.g., a brief session with a physician or a nurse during regular cardiac consultation). In addition, if indeed improved mental well-being mediates the effect of social support on health behavior and cardiovascular risk as suggested above, then incorporating social support in intervention design could increase intervention success.

Our results showed that less than 1% of the surveyed patients reported full compliance with lifestyle recommendations for patients diagnosed with CHD and almost half (48%) reported three or more unhealthy behaviors. These results are in accordance with research in the healthy Spanish population showing that less than 1% of individuals comply with lifestyle recommendations regarding cardiovascular health (nonsmoking, normal weight, physical activity at goal, and healthy diet) [43]. The low vegetable consumption and low physical activity are also in accordance with results from the general Spanish population, as are the generally high adherence to fish consumption and avoidance of sugary drinks [43]. A study in older Spanish adults (≥60 years old) found that 43% were completely sedentary and 54% reported only occasional light physical activity mirroring the results found in the current study [44]. Comparing the results to those of other samples of CHD patients, the current sample had a lower prevalence of smoking (10%) and obesity (26%) compared to the average reported by patients from 24 European countries (16% and 38%, respectively) in the EUROASPIRE IV study [4].

Regarding socio-demographic factors, we found no differences in unhealthy behaviors as a function of marital status. These results are inconsistent with previous findings showing that married individuals have a better risk profile and better cardiovascular health [9]. However, we found differences between men and women, in accordance with previous results showing that men are less likely to follow advice regarding lifestyle changes (e.g., diet and smoking) [7]. Finally, we also found that older patients report fewer unhealthy behaviors.

A notable strength of the current study is the wide age range of the sample and the possibility to extrapolate the results to the general patient population. Limitations that need to be taken into account include the possibility of selection biases (e.g., not participating in the survey due to illness) and inaccurate knowledge of participants regarding the condition diagnosed. The specific clinical diagnoses of participants and their exact timing were not recorded.

Issues related to the measurement of the unhealthy behaviors or cardiovascular risk factors could have an impact on the results. In particular, for some behaviors (e.g., physical activity) the survey did not contain sufficient detail to evaluate adherence according to the specific European Guidelines and no information was available for some aspects part of a healthy diet according to the Guidelines (e.g., unsalted nuts or salt consumption). For other behaviors (e.g., fast food and sugary drinks consumption) there are no specific guidelines but a general recommendation against their consumption on a regular basis, thus, we used our expert judgment to set the criterion for these variables. There was no detailed clinical information available regarding the traditional risk factors and no information about previous personal or family history of CHD. In addition, we could not control for patients’ lifestyle before the diagnosis and did not examine medication adherence. The latter is an especially important part of secondary prevention efforts that could have even stronger effects than lifestyle factors [4].

Given the correlational nature of this research, a bidirectional relationship is also possible, such that the inability of patients to adopt a healthier lifestyle could also be contributing to lower mental health. For instance, individuals who do not engage regularly in physical activity are more likely to suffer low moods and depression [45]. Finally, we adopted a behavioral approach and gave equal weight to all unhealthy behaviors. Future studies should consider alternative risk scores, as some behaviors may be more harmful than others (e.g., smoking may increase risk to a larger extent than does lack of physical activity). In addition, it would be of interest for future research to investigate unhealthy behaviors and the documented relationships comparing patients with CHD to patients with other chronic diseases or a healthy population.

Finally, another psychosocial factor that could be of interest is adverse working conditions such as job strain and small decision latitude that have been related to the development of CHD [46] Unfortunately in the current sample the large majority of patients (67%) were retired and no information about their previous working conditions was available. It is possible that long-lasting unfavorable work conditions contribute to unhealthy behavior patterns and this way increase the risk of CHD. However, findings regarding the relationship between stressful work conditions and unhealthy behaviors appear to be inconclusive and more research on this issue is needed [47].

## Conclusions

Using recent data, the current study confirms that there is a need for individual or community-based interventions aiming to increase adherence to lifestyle recommendations among CHD patients. Low mental well-being (i.e., problems with coping, low self-esteem, and perceived stress) was associated with more unhealthy behaviors, with an effect size of clinical significance (i.e., doubling the odds of accumulated unhealthy behaviors). This relationship was even stronger in patients with obesity. These results suggest that a large proportion of patients may not adhere to lifestyle recommendations not because they purposefully choose to do so but because they have difficulty coping with the perceived barriers to introducing and maintaining the recommended healthy behaviors. This possibility should be investigated further using prospective research designs. These findings also speak to the potential utility of further research on brief interventions that provide coping resources such as action and coping planning instructions to help patients adopt a healthier lifestyle.

## Supporting information

S1 STROBE checklist (DOC)
